# Orientin mediates protection against MRSA-induced pneumonia by inhibiting Sortase A

**DOI:** 10.1080/21505594.2021.1962138

**Published:** 2021-08-09

**Authors:** Li Wang, Shisong Jing, Han Qu, Kai Wang, Yajing Jin, Ying Ding, Lin Yang, Hangqian Yu, Yan Shi, Qianxue Li, Dacheng Wang

**Affiliations:** a College of Animal Science, Jilin University, ChangchunChina; b Key Laboratory of Jilin Province for Zoonosis Prevention and Control, Changchun Veterinary Research Institute, Chinese Academy of Agricultural Sciences, Changchun, China; c School of Pharmaceutical Science, Jilin University, ChangchunChina

**Keywords:** *Staphylococcus aureus*, anti-virulence, orientin, inhibitor, Sortase A, pneumonia

## Abstract

Drug-resistant pathogenic *Staphylococcus aureus* (*S. aureus*) has severely threatened human health and arouses widespread concern. Sortase A (SrtA) is an essential virulence factor of *S. aureus*, which is responsible for the covalent anchoring of a variety of virulence-related proteins to the cell wall. SrtA has always been regarded as an ideal pharmacological target against *S. aureus* infections. In this research, we have determined that orientin, a natural compound isolated from various medicinal plants, can effectively inhibit the activity of SrtA with an IC_50_ of 50.44 ± 0.51 µM. We further demonstrated that orientin inhibited the binding of *S. aureus* to fibrinogen and diminished biofilm formation and the attaching of Staphylococcal protein A (SpA) to the cell wall *in vitro*. Using the fluorescence quenching assay, we demonstrated a direct interaction between orientin and SrtA. Further mechanistic studies revealed that the residues Glu-105, Thr-93, and Cys-184 were the key sites for the binding of SrtA to orientin. Importantly, we demonstrated that treatment with orientin attenuated *S. aureus* virulence of *in vivo* and protected mice against *S. aureus*-induced lethal pneumonia. These findings indicate that orientin is a potential drug to counter *S. aureus* infections and limit the development of drug resistance.

## Introduction

*Staphylococcus aureus* (*S. aureus*) has the potential to induce pathogenicity and can cause various community and hospital-acquired infections [1]. This pathogen usually causes superficial infections of the skin and soft tissue, infections from surgical instruments, and sometimes fatal bacteremia and pneumonia [2,3]. The importance of *S. aureus* has been highlighted by the emergence and spread of highly virulent, multidrug-resistant *S. aureus*, especially methicillin-resistant *S. aureus* (MRSA), which is characterized by significant morbidity, mortality, and high financial costs, thereby seriously endangering public health [4]. MRSA has a remarkable ability to acquire resistance to a wide range of antibiotics. Therefore, the treatment of MRSA infection has become more challenging for clinicians, thus necessitating the development of new strategies to combat MRSA infections^5^.

*S. aureus* can express a variety of virulence determinants, which can escape host immune response and cause a series of diseases [5]. Therefore, targeting virulence is an alternative method to treat MRSA infections. In *S. aureus*, SrtA is found to cleave between the threonine and glycine of the LPXTG motif, and covalently anchored the protein to the bacterial cell wall via a transpeptidation reaction [6,7]. Surface proteins such as protein A, clumping factor proteins, collagen adhesion protein, pili proteins, and fibronectin-binding proteins [8] are all requiring SrtA to attach to the cell wall by this type of reaction [9]. In addition, these surface proteins anchored by SrtA exert important effects on bacterial adhesion, immune escape, host tissue invasion, and biofilm formation by suppressing phagocytosis and opsonization [10,11]. Without these functional proteins, most pathogens would not be able to sustain infection [11]. It has been reported that *S. aureus* mutants lacking SrtA are devoid of surface proteins and cannot induce abscess within organ tissues or give rise to fatal bacteremia after being injected in the mouse bloodstream [12,13]. Therefore, SrtA has been recognized as an optimal target for designing novel drugs against *S. aureus* infections by disrupting the adhesion of bacterial virulence and biofilm formation without affecting the bacterial viability [10,14,15].

Previously reported SrtA inhibitors include natural products [9,16–18], synthetic products [19,20], and designed peptidomimetic compounds [21,22]. Orientin, a flavonoid isolated from various medicinal plants, is widely used in medicine because of its anti-inflammatory, antioxidant, and antitumor effects [23–26]. In this study, we observed that it was an effective inhibitor of SrtA. Furthermore, the protective effect of orientin on MRSA-induced lethal pneumonia in mice was assessed, which indicated that orientin can be developed as a potential anti-MRSA drug.

## Materials and methods

### Bacteria, chemicals, and growth conditions

LAC, the strain of *S. aureus* USA300, was provided by the American Type Culture Collection (Manassas, VA, USA). The mutant with SrtA deletion (Δ*srtA*) was preserved in our laboratory. *E. coli* BL21(DE3) was used as the host to express the protein and was purchased from TaKaRa Biological Company (Dalian, China). Abz-LPATG-Dap (Dnp)-NH_2_ (Abz: ortho-aminobenzoic acid; Dnp:2,4-dinitrophenyl), a peptide substrate, was purchased from LifeTein (Beijing, China). The rabbit anti-SrtA polyclonal antibody was prepared by our team. The orientin (purity >98%) was purchased from Sigma–Aldrich. Other chemical reagents were provided by Sangon Biotech (Shanghai, China). The *S. aureus* was routinely cultured in brain-heart infusion broth (BHI, Solarbio, Beijing, China) at 37°C.

## Cloning, expression, and purification of SrtA and its mutants

The sequence of *srtA* from *S. aureus* USA300 was retrieved from the GenBank database. The *srtA* gene lacking the transmembrane domain (N_1–59_) was amplified using PCR. The PCR product was then digested and cloned in the BamHI/XhoI restriction sites of the pET28a vector, yielding pET28a-*srtA*. The site-directed mutagenesis of T93A-SrtA, E105A-SrtA, and C184A-SrtA was conducted using pET28a-*srtA* using a Multi-Site Mutagenesis Kit (Transgen, Beijing, China). All the primers are presented in Table 1. The expression vector was then transformed into the BL21(DE3) expression host, and the bacteria were cultured in BHI medium supplemented with kanamycin (50 µg/mL) at 37°C. In addition, isopropyl-β-D-thiogalactoside (1 mM) was used to induce recombinant SrtA for 4 h at 16°C. Whole-cell lysates of bacteria were prepared through ultrasonic crushing. Recombinant SrtA_ΔN59_ or its mutants were purified using the 6 × His/Ni-NTA system refer to a previous study [27].

**Table 1.** Primers used in this study

Primer name	**Sequences (5ʹ-3ʹ)**
*srtA*-F	GGGAATTCCATATGCAAGCTAAACCTCAAATTCCG
*srtA*-R	CGCGGATCCTTATTTGACTTCTGTAGCTACAAAGA
T93A-*srtA*-F	GACCAGCAGCACCTGAACAATTAAA
T93A-*srtA*-R	CTGGATATACTGGTTCTTTAATATCAGC
E105A-*srtA*-F	GCTTTGCAGCAGAAAATGAATCAC
E105A-*srtA*-R	TTACACCTCTATTTAATTGTTCAG
C184A-*srtA*-F	TACTGCTGATGATTACAATGAAAAG
C184A-*srtA*-R	ATTAATGTTAATTGTTTATCTTTAC

## Fluorescence resonance energy transfer assay-based screening of SrtA inhibitor

Fluorescence resonance energy transfer (FRET) was detected the activity of orientin against SrtA, as previously described [28]. The reaction mixture (300 µL) consisted of the reaction buffer (50 mM Tris-HCl, 5 mM CaCl_2_, 150 mM NaCl, pH 7.5), 4 µM purified SrtA, and various concentrations of orientin (0–200 µM). The sample was incubated at 37°C for 1 h, followed by the addition of substrate peptide (10 μM). After incubation for another hour at 37°C, the fluorescence intensity value was detected at the excitation and emission wavelengths of 350 nm and 495 nm, respectively.

## Reversible inhibition assay of SrtA

The 10-fold IC_50_ concentration of orientin was incubated with 100 µL of SrtA (150 µM) for 1 h at 37°C, followed by the addition of 9.9 mL reaction buffer. Then, 190 µL of the mixture was added to each well, and substrate peptide (10 μL) was added until a final concentration of 10 μM was achieved. A multifunctional microplate reader was used to record the fluorescence intensity at 350 nm for excitation and 495 nm for emission.

## Susceptibility testing and growth curve assay

The broth microdilution method was performed to determine the MIC of orientin for *S. aureus* USA300 as previously described [29]. Briefly, orientin was diluted two-fold serially in a 96-well plate at concentrations ranging from 2 to 1024 µg/mL, followed by inoculation with *S. aureus* USA300 (10^6^ CFU/mL) and incubation at 37°C for 16 h. After incubation, the absorbance (OD) value at 600 nm was measured. For the growth curve experiment, the overnight bacterial culture was diluted in fresh BHI (1:100) with various concentrations of orientin (0–200 µM). *S. aureus* Δ*srtA* was used as the control group. Each sample was cultured at 37°C, and the OD_600_ was measured at 1 h intervals.

## Cytotoxicity assay

Cytotoxicity was determined using the Cell Counting Kit-8 (CCK-8) as previously described [30]. Briefly, 100 µL Vero cells (5 × 10^4^ cells/well) were seeded in a culture plate, followed by 24 h incubation at 37°C and under 5% CO_2_. Then, the original medium was removed gently, and the freshly prepared medium containing various concentrations of orientin (0–400 µM) or DMSO was added to the cells. Afterward, 10 µL of the CCK-8 solution was carefully added to each well and incubated for another 4 h in an incubator. The OD value at 450 nm was measured for assessing the cell viability. The experiment was repeated at least thrice, and the curve of the orientin concentration versus the cell viability was drawn using the statistical program GraphPad Prism version 8.0.

## Adherence of *S. aureus* to immobilized fibrinogen

The *S. aureus* USA300 was grown in BHI broth for 12 h and then diluted (1:100) in fresh BHI containing different concentrations of orientin, then continued to culture until an OD_600_ reached 0.5. The ∆*srtA* mutant was cultured under the same conditions as a positive control. Subsequently, the bacterial culture was added to a 96-well plate previously coated with bovine fibrinogen (20 µg/mL). Then, the sample was incubated for 2 h at 37°C, and 25% (v/v) formaldehyde was added to fix the adherent bacterial cells for 30 min after discarding the suspension. The formaldehyde was removed, and the plate was washed twice with PBS, following which, crystal violet was added to stain the cells for 20 min. The wells were gently washed with PBS and dried, and the OD value at 570 nm was measured.

## Crystal violet biofilm assay

After overnight culturing, *S. aureus* was diluted using fresh BHI by 1:100, and different concentrations of orientin ranging from 0 to 200 μM were added with shaking at 37°C to OD_600_ of 0.6. The bacterial culture (5 µL) was then added to BHI broth containing 1% glucose to a final volume of 200 µL and continue cultured for 18 h. Then, the medium was discarded carefully and washed three times with PBS. Then, the biofilms were stained with a 0.1% (w/v) crystal violet solution for 20 min at ambient temperature conditions. The crystal violet solution was discarded, and the wells were washed thrice with sterile PBS. After drying the plates, 95% ethanol was added into each well, and the absorbance at 570 nm was measured.

## FITC-IgG binding to Staphylococcal protein A (SpA)

Overnight culture of *S. aureus* and *S. aureus* Δ*srtA* was diluted 1:1000 in TSB medium, and different concentrations of orientin (0–200 µM) were added to the culture with shaking at 37°C until the logarithmic growth phase. The bacteria collected by centrifugation were washed thrice with PBS, after which the bacterial precipitate was resuspended with 50 µL of 1:200 diluted FITC-labeled rabbit IgG (Solarbio, Beijing, China). Then, the bacterial precipitate was incubated at ambient temperature for 30 min, and the harvested bacteria were rinsed thrice by PBS. Then, the bacteria were resuspended in 100 µL PBS and added to a black 96-well plate. The Multimode Microplate Reader (Tecan, Spark 20 M) was used to measure the fluorescence intensity at 490 nm for excitation and 520 nm for emission.

## Western blot analysis

An equal amount of protein extract was separated using SDS-PAGE, followed by transfer to the polyvinylidene difluoride (PVDF) membrane (GE Healthcare, UK). The membrane was incubated in 5% BSA overnight at 4°C. After washing with TBST (TBS + 1‰ Tween 20), the membrane was incubated with rabbit anti-SrtA polyclonal antibody (1:3000) at room temperature. After incubation for 2 h, the membrane was washed thrice and incubated with HRP-conjugated goat anti-rabbit IgG (diluted 1:10,000 in TBST) for 2 h. After washing thrice, the membrane was incubated with Super ECL Plus (US EVERBRIGHT, Suzhou, China) and visualized in an ECL detection system (GE Healthcare, UK). The cytoplasmic protein ClpP was treated in the same way as an internal control. Band quantification was performed using the software ImageQuant TL.

## Fluorescence quenching assay

The binding constants (*K_A_*) of orientin to SrtA were determined using the fluorescence quenching assay. The spectra were recorded in the wavelength interval of 280–400 nm. The protocols used to perform the measurements have previously been described [31–33].

## Molecular modeling of SrtA-orientin interactions

For molecular docking simulations, the crystal structure of SrtA (PDB code: 1T2P) was obtained from Protein Data Bank, and the 3D structure of orientin was also constructed using the software Hyperchem version 8.0 (Hypercube, Inc.). Standardized docking of SrtA-orientin was determined using the software packages AutoDock vina 1.1.2 [34] and Amber 14 [35,36]. The detailed procedure for simulating the molecular dynamics and calculating the binding free energy is described in earlier studies [37,38].

## Invasion assay

The A549 cells were routinely sub-cultured at 37°C with 5% CO_2_. Cells in the logarithmic growth phase were seeded in a 24-well plate at a density of 2.5 × 10^5^ per well and cultured for 20 h. *S. aureus* was mixed with orientin ranging from 0 to 100 µM and cultured at 37°C until an OD_600_ of 1.0 was reached. Then, the cell culture medium was discarded, and the cells were resuspended in DMEM medium. After that, a bacterial suspension containing 2 × 10^7^ CFU/mL was added to each well. After incubation for 2 h, 300 µg/mL gentamicin was added to stop the invasion. Then, the cells were lysed after washing with sterile PBS and spread on BHI agar plates to calculate the number of colonies in each sample.

## Pneumonia model experiment

The pneumonia model was induced as previously described [39,40] in 7-week-old female C57BL/6 J mice. For survival experiments, a group of 10 mice was infected with 30 µL of *S. aureus* culture (2 × 10^8^ CFUs) via the intranasal route. An hour after infection, the mice were intraperitoneally injected with orientin (100 mg/kg) at intervals of 12 h. Similarly, mice in the control group were injected with sterile PBS containing 0.5% DMSO. The mice were monitored every 12 h for 96 h after administration to calculate the survival rate. For estimation of the bacterial count in the lung tissue and histopathological analysis, the mice were infected with 30 µL (1 × 10^8^ CFUs) of *S. aureus* cultures, and the infection was allowed to progress for 2 d. The mice were then sacrificed, and the lungs were collected, weighed, and homogenized. Then, appropriate dilutions were plated on BHI-agar plates until a single colony appeared and counted. The lung tissues of mice in each group were aseptically separated and fixed in 10% formalin. The lung tissue sections were observed under an optical microscope after conventional hematoxylin and eosin (H&E) staining.

## Statistical analysis

The data were presented in the manner of mean ± SD, and values of *P* < 0.05 were considered statistically significant. All the statistical analyses in this study were conducted using the statistical program GraphPad Prism version 8.0.

## Results

### Identification of orientin as an SrtA inhibitor

**Figure 1.**
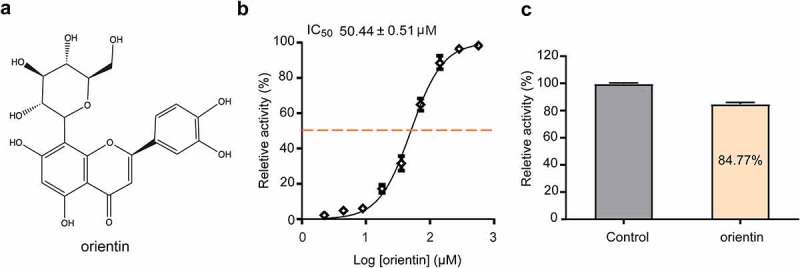
Orientin as a reversible inhibitor of SrtA

FRET is the main method for the screening of SrtA inhibitors based on SrtA cleavage of the LPXTG peptide [20,41]. In this experiment, we observed that orientin (Figure 1a) inhibited the activity of SrtA in a dose-dependent manner, with an IC_50_ value of 50.44 ± 0.51 µM (Figure 1b). Then, SrtA was incubated with 10-fold IC_50_ of orientin to determine whether the binding was reversible. The activity of SrtA was observed to be recovered by 84.77 ± 1.28%, indicating that orientin was a reversible inhibitor of SrtA (Figure 1c).

(a) Chemical structure of orientin. (b) Determination of the IC_50_ values of orientin that inhibit the cleavage of Abz-LPATG-Dap (Dnp)-NH_2_ substrate, using the FRET assay. (c) SrtA was treated with 10 × IC_50_ of orientin and then diluted, and its activity was measured by FRET assay. Untreated SrtA (Control) was considered as 100% activity.

## MIC, growth curve, and cytotoxicity of orientin

**Figure 2.**
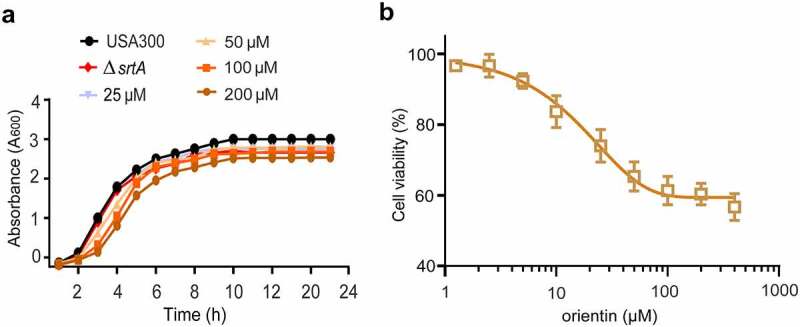
Growth curve and cytotoxicity of orientin

Drug safety is highly important for its further development and application. The results of the MIC and growth curve indicated that the MIC of orientin was greater than 512 µg/mL, and 200 µM of orientin was found to have little inhibitory effect on *S. aureus* growth (Figure 2a). Importantly, when orientin was incubated with Vero cells for 24 h, there was no cytotoxicity at 200 µM of orientin (Figure 2b). These data demonstrate that orientin could be further developed as a potential SrtA inhibitor due to its safety and high efficiency.

(a) Growth curves of *S. aureus* USA300 treated with different concentrations of orientin (0–200 µM), with Δ*srtA* as the positive control. (b) Percent cell viability of Vero cells measured by CCK-8 assay after 24 h of incubation with the tested concentrations of orientin (0–400 µM).

## Effect of orientin on the adhesion of *S. aureus* to fibrinogen

**Figure 3.**
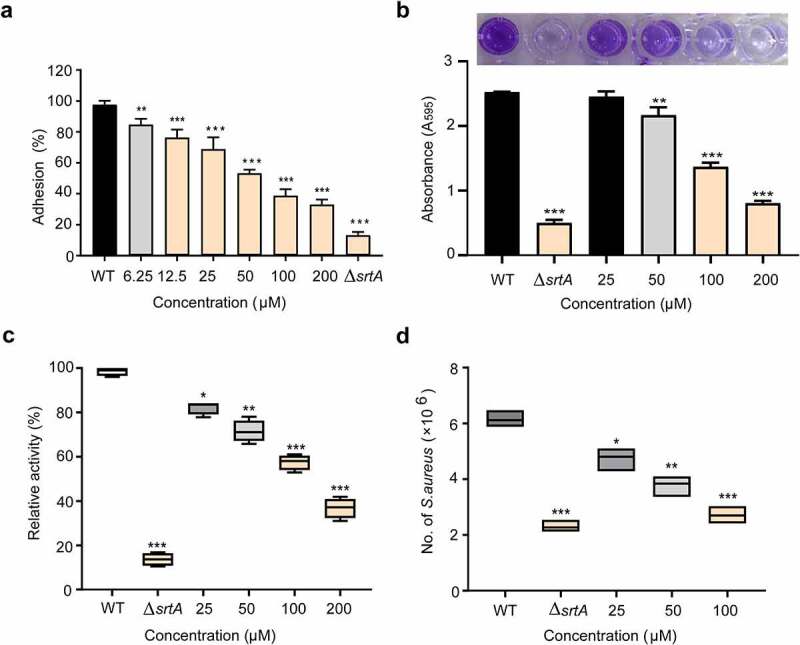
Effect of orientin inhibitors on virulence-related phenotypes in *S. aureus.*

Given SrtA mediates anchoring of several adhesion-related proteins, such as ClfA/ClfB and binding fibronectin (FnBPs), to the cell wall surface [42]. We further investigated the effect of orientin on the adhesion of *S. aureus* to fibrinogen. As presented in Figure 3a, orientin markedly suppressed *S. aureus* from adhering to fibrinogen (*P* < 0.001). The wild-type (WT) group treated with 200 µM orientin had a significantly inhibitory ability of adhesion to fibrinogen was 33.10 ± 1.41%. The Δ*srtA* group showed that the fibrinogen was 13.30 ± 0.92%.

(a) Impact of orientin on the adherence ability of *S. aureus* onto fibrinogen. (b) Crystal violet staining biofilm assay to determine the effect of orientin on the biofilm formation of *S. aureus*. (c) Fluorescence intensity analysis of the *S. aureus* protein A (SpA) stained with FITC-labeled rabbit IgG. (d) Orientin inhibits the internalization of *S. aureus* into A549 cells. A549 cells were infected with *S. aureus* pre-treated with various concentrations of orientin. Then cells were lysed 2 h after infection and the number of surviving *S. aureus* within the cells was determined through serial dilutions onto the LB agar plates. Error bars indicate the mean ± SD of triplicates. * *P* < 0.05, ** *P* < 0.01, *** *P* < 0.001 vs. WT group upon Student’s t-test (two-tailed)

## Effect of orientin on the biofilm formation

Biofilm formation is an important cause of antibiotic resistance and chronic biofilm-associated infections caused by *S. aureus*. It often leads to significant increases in morbidity and mortality [43]. Therefore, effective reduction of the biofilm formation is highly significant. Since SrtA-mediated surface proteins are closely related to the formation of biofilm, we further examined the effect of orientin on biofilm formation by the crystal violet staining assay. Figure 3b shows that orientin inhibited the formation of *S. aureus* biofilm. Compared to the WT group (untreated *S. aureus*), the biofilm biomass was significantly decreased to 31.90 ± 0.25% upon exposure of the *S. aureus* strain to 200 µM orientin (*P* < 0.001), whereas the biofilm biomass of Δ*srtA* was only 19.76 ± 0.13%.

## Effect of orientin on the anchoring of SpA

*S. aureus srtA* mutants cannot anchor proteins to the cell wall [44]. Therefore, we analyzed the effect of orientin on the anchoring of SpA. One of the outstanding characteristics of SpA is that it can specifically bind to the FITC-labeled IgG of several mammalian species, the abundance of IgG binding to SpA in the bacterial cell wall envelope can be evaluated roughly from the fluorescence intensity [45]. As showed in Figure 3c, the WT group was observed to show a stronger fluorescence intensity. When *S. aureus* was treated with 200 µM orientin, significantly lower fluorescence was observed, and the relative activity was only 37.32 ± 1.84% compared to the WT group (*P* < 0.001). These results indicated that orientin inhibited the anchoring of SpA to bacterial cell wall by suppressing SrtA.

## Effect of orientin on the *S. aureus* internalization

In *S. aureus*, adhesion to and invasion of host cells mediated by the surface proteins are the major virulence strategies for immune evasion and survival [46]. Therefore, the inhibition of SrtA of *S. aureus* by employing strong inhibitory compounds or the deleting of the *srtA* gene interferes with the bacterial invasion ability, and thus attenuates the bacterial virulence [14,44]. As expected, the WT group (untreated *S. aureus*) exhibited a stronger ability to invade A549 cells, and this ability decreased significantly when *S. aureus* was treated with 100 µM of orientin (*P* < 0.001). Thus, orientin could effectively inhibit *S. aureus* internalization by inhibiting the SrtA (Figure 3d).

## Effect of orientin on the expression of SrtA

**Figure 4.**
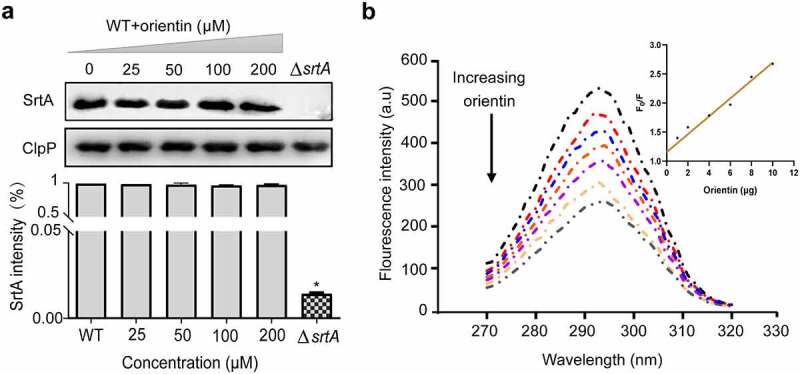
Determination of the effect of orientin on the expression level of SrtA and the interaction between orientin and SrtA using the fluorescence quenching assay

To further evaluate whether orientin could inhibit the expression of SrtA, Western blot was performed. The addition of different concentrations of orientin (0, 25, 50, 100, or 200 µM) was observed not to affect the expression of SrtA (Figure 4a) compared to the WT group (untreated *S. aureus*). This implied that orientin could effectively inhibit the activity of SrtA but not its expression.

(a) Western blot analysis of SrtA from *S. aureus* treated with various concentrations of orientin (0–200 µM). (b) Emission spectra of SrtA in the presence of different concentrations of orientin at λex = 280 nm. Inset: Stern-Volmer plot describing the SrtA quenching caused by association with orientin.

## Determination of the interaction of orientin with SrtA

A fluorescence quenching experiment was used to evaluate the interaction between orientin and SrtA. The change in the intensity of fluorescence emission was measured within 1 min after the addition of orientin (0–12 µg) to SrtA. It was observed that orientin gradually quenched the fluorescence of SrtA in a dose-dependent manner compared to free SrtA (Figure 4b), and F_0_/F was found to be linearly dependent on the quencher level (Figure 3b, inset). We further determined the binding constant *K_A_* of SrtA to orientin to be 7.12 × 10^4^ l/mol, indicating a direct binding interaction between orientin and SrtA.

## Determination of the molecular mechanism of the interaction between orientin and SrtA

**Figure 5.**
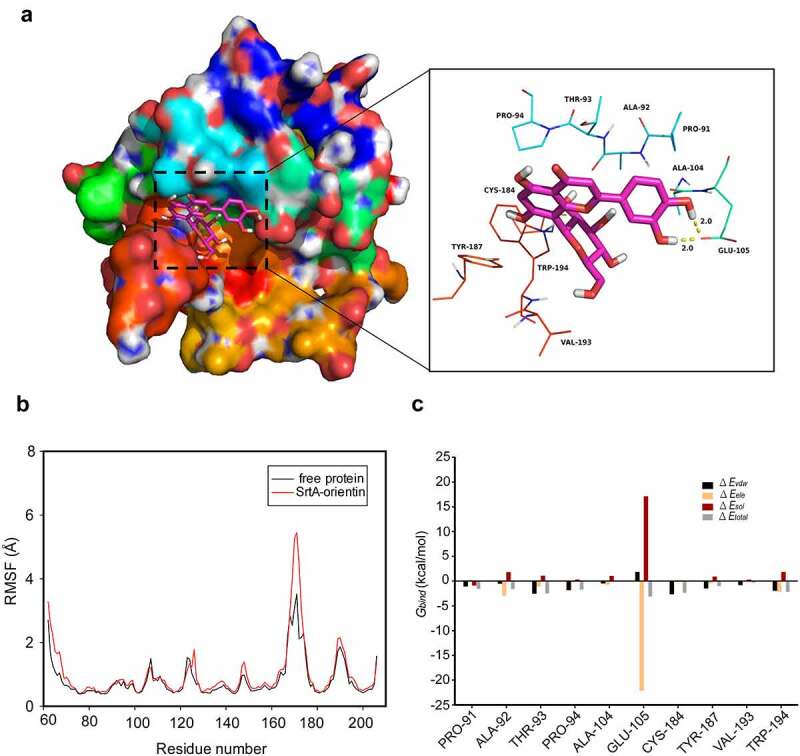
Molecular modeling revealed the interaction between orientin and SrtA

To further clarify the mechanism of interaction between orientin and SrtA, a molecular modeling study was carried out. In the SrtA-orientin complex, residue Glu-105 exhibited a strong electrostatic (∆*E_ele_*) contribution of < –22.0 kcal/mol (Figure 5c). Further analysis revealed that the residue Glu-105 was close to the hydroxyl group of orientin, forming a double hydrogen bond interaction with a length of 2.0 Å and 2.0 Å (Figure 5a). Moreover, the residue Thr-93 made a considerable van der Waals force contribution (∆*E_vdw_* < －2.5 kcal/mol) (Figure 5c), which was due to the proximity between the residue Thr-93 and orientin (Figure 5b). Except for Thr-93 residues, most of the energy contribution of residues (including Pro-91, Ala-92, Pro-94, Cys-184, Val-193, and Trp-194) could be ascribed to van der Waals forces mostly through the hydrophobic interactions.

(a) Binding free energy decomposition of each residue between orientin and modeled *S. aureus* SrtA. (b) The root-mean-square fluctuation (RMSF) (Å) graph of free-SrtA (black) and SrtA-orientin (red) complex during the 40-ns molecular modeling (MD). (c) Decomposition of the binding energy on a per-residue basis in the SrtA-orientin complex.

Based on the results of molecular modeling of the interactions between orientin and SrtA, we conducted site-directed mutagenesis of the amino acids of SrtA which may interact with orientin. Then, fluorescence quenching assays were used to evaluate the binding affinity of SrtA and its mutants (Thr-93, Glu-105, and Cys-184) to orientin. As is shown in Table 2, the binding constant (*K_A_*) between SrtA mutants (Thr-93, Glu-105, and Cys-184) and orientin was markedly lower than that of WT SrtA (*P* < 0.05 or *P* < 0.01), indicating that residues Thr-93, Glu-105, and Cys-184 were the critical sites for the binding of orientin to SrtA.

**Table 2.** The values of the binding constants (*K_A_*) based on fluorescence quenching assay

**Proteins**	**WT-SrtA**	**D170A**	**E105A**	**C184A**
*K_A_* (1 × 10^4^) l/mol	7.12	5.37*	4.25*	3.12**
n	0.9861	0.9741	0.9482	0.8847

*P < 0.05, ** *P* < 0.01 compared with the WT-SrtA group.

## *In vivo* protection orientin on MRSA-induced pneumonia

**Figure 6.**
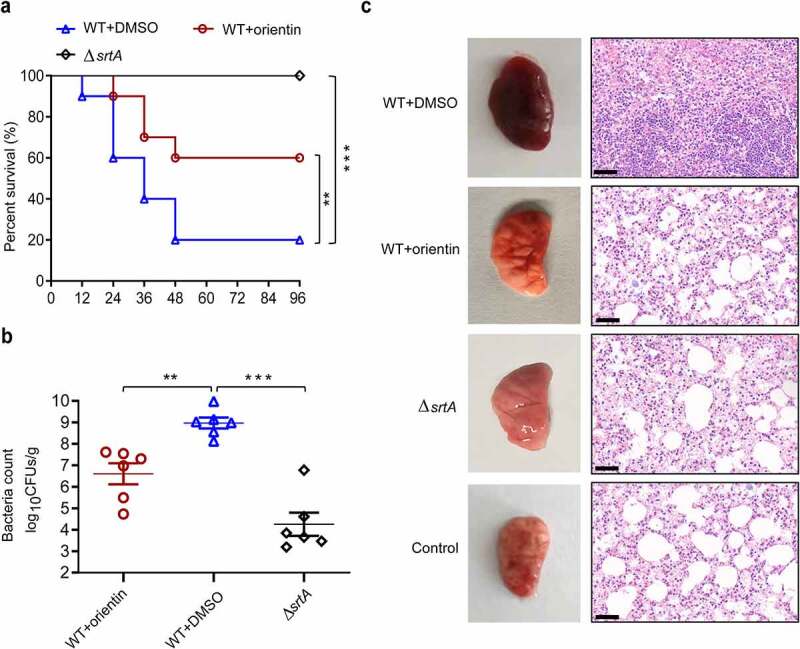
The therapeutic and protective effects of orientin on mice

To evaluate the therapeutic activity of orientin in the lung, mice were challenged with a lethal dose of *S. aureus* (2 × 10^8^ CFU per mL) and then treated with 100 mg/kg orientin. The untreated mice were observed to die at 12 h after intranasal inoculation with *S. aureus*, and the survival rate was 20% within 72 h (Figure 6a). However, mice treated with 100 mg/kg orientin showed a significantly improved survival rate of 60% within 72 h (*P* < 0.01). The results of bacterial load assay showed that orientin treatment significantly reduced the number of viable *S. aureus* in the lung tissue of mice, compared to the untreated group (*P* < 0.001) (Figure 6b).

(a) Effect of orientin treatment on the survival of mice (n = 10) infected with a lethal dose of *S. aureus*. WT+DMSO (control) vs. orientin-treated ** indicates *P* < 0.01. (b) Effect of orientin treatment (100 mg/kg) on bacterial load in lungs of mice (n = 6). WT+DMSO (control) vs. orientin-treated ** indicates *P* < 0.01, *** *P* < 0.001. A two-tailed Mann–Whitney U-test was used for comparison. Horizontal bars represent the means. (c) Gross pathological changes and histopathology of the lungs of mice treated with orientin (100 mg/kg/d) or untreated mice. Scale bar, 50 µm. The animal data were obtained from two separate experiments.

To evaluate the potential therapeutic effects of orientin treatment, the histology of lung tissue was examined. The lung tissues of orientin-treated mice were observed to be pink and spongy with less local infection, whereas those of mice in the infected control group were dark red. Histopathological examination revealed that the lungs of infected mice without treatment had a certain degree of acute injury, which was characterized by hyperemia and edema in the interstitial region with marked focal hemorrhage. After treatment with 100 mg/kg orientin, inflammatory symptoms were significantly reduced, and a small number of inflammatory cells infiltrated (Figure 6c). Overall, these results indicate that orientin can attenuate the virulence of *S. aureus in vivo* and protect mice from lethal *S. aureus*-induced pneumonia.

## Discussion

Drug-resistant bacteria such as MRSA are becoming increasingly common and are causing a global public health crisis [47,48], MRSA can cause soft tissue infections, pneumonia, systemic infection, septic shock syndrome, and other diseases [49]. Compared to methicillin-susceptible *Staphylococcus aureus* (MSSA), MRSA has strong pathogenicity, high infection rate, and high mortality, which makes it the primary harmful drug-resistant pathogen worldwide [50]. The recurrent epidemics of drug-resistant *S. aureus* reveal a rapid decline in the efficacy of antibiotics, with the emergence of resistance to these drugs, the choice of anti-MRSA drugs may be further reduced in the future. Therefore, anti-virulence therapy is highly significant for counter *S. aureus* infections and avoiding the development of drug resistance [51–53]. Moreover, anti-virulence therapy can avoid the long-standing side effects of antibiotics, that is, drugs can kill disease-causing bacteria while killing beneficial and commensal bacteria which are vital in promoting the development of the immune system, metabolism, and resistance to pathogen colonization in the human body [52,54,55].

SrtA is a major virulence factor involved in covalently anchoring the surface proteins onto the cell wall of Gram-positive bacteria [11] and is regarded as an ideal anti-virulence target. These surface proteins in *S. aureus* can recognize the adhesive matrix molecules, which play an important role in biofilm formation, bacterial adhesion, immune escape, and host tissue invasion [56].

Natural compounds have become attractive anti-infection agents due to their safety and environmental friendliness recognized by long-term practice [57]. In the present study, we selected Chinese herbs-derived natural compounds based on the FRET assay as the SrtA inhibitors. Orientin was identified as a candidate for the inhibition of SrtA *in vitro* with an IC_50_ of 50.44 ± 0.51 µM. In addition, the IC_50_ value of orientin against *S. aureus* SrtA *in vitro* was significantly lower than that of natural product inhibitors reported previously, such as quercetin, kaempferol, galangin, and isorhamnetin [9], which means that orientin may be more effective in inhibiting SrtA. In addition, inhibitors of SrtA can be categorized as either covalent or non-covalent [58,59]. If there is a choice, drug developers prefer non-covalent inhibitors to covalent modify enzyme inhibitors, which overcome the drawbacks of covalent inhibitors, such as high toxicity, non-recoverability, non-repairability, and other side effects [60]. In the present study, orientin as a non-covalent inhibitor which is reversibly binds to SrtA. In addition, the safety of a drug is the primary condition for its further development and application, we found that orientin can effectively inhibit the activity of SrtA without affecting *S. aureus* growth and Vero cells at the concentration required to inhibit SrtA. Thus, orientin can be a promising candidate inhibitor of SrtA due to its high efficiency and low toxicity and can be analyzed further in subsequent studies. Furthermore, orientin can significantly inhibit bacterial adherence onto fibrinogen, and suppress SpA anchoring to the cell wall. Bacteria can proliferate and colonize on biotic or abiotic surface by forming a biofilm, which can protect bacteria against the host immune system and antibiotic treatment [61]. When orientin was co-incubated with *S. aureus*, the biofilm formation was decreased. It was previously reported that a mutant with a deletion of the FnbA and FnbB genes did not express fibronectin-binding proteins FnBPA and FnBPB and lacked the ability to adhere to fibronectin or to form biofilm [62]. Therefore, the inhibition of orientin on biofilm formation may be due to the adhesion of fibronectin-binding proteins mediated by SrtA. In addition, the accumulation of bacteria during biofilm formation is also related to the polysaccharide intercellular adhesin (PIA), which is encoded by ica operon [63].

Importantly, orientin reduced the adhesion-dependent invasion of A549 cells by *S. aureus*. This result may also be related to orientin can also affect FnBP_S_, which is involved in bacterial entry into non-phagocytic mammalian cells by forming a molecular bridge between Microbial Surface Components Recognizing Adhesive Matrix Molecules (MSCRAMMs) and integrins on the host cell [64]. Subsequently, the results of molecular modeling suggested that the binding of orientin to the binding pocket of SrtA mainly depends on the hydrogen bonding, electrostatic, and van der Waals interactions. Using fluorescence quenching assay, the residues Glu-105, Thr-93, and Cys-184 were confirmed to be the key sites for the binding of SrtA to orientin. In SrtA, the active site Cys-184 is involved in the process of catalysis and can attack the T-G peptide bond of the protein containing LPXTG to form an enzyme-acyl intermediate [6,65]. When Cys-184 is mutated to glycine, the transpeptidation activity of the SrtA would be lost [66]. Fluorescence quenching assay verified the binding of orientin to the active site Cys-184. Moreover, the *in vivo* protective effect of orientin on MRSA-induced lethal pneumonia was identified in mice. The significant anti-virulence effects of orientin *in vivo* and *in vitro* indicate that orientin can be developed as a potential anti-MRSA drug.

## Supplementary Material

Supplemental Material

## Data Availability

The data that support the findings of this study are available from the corresponding author.
